# Supplementary figures and images for: Exploring the transmission of cognitive task information through optimal brain pathways
Source: PLoS Comput Biol. 2025 Mar 7;21(3):e1012870. doi: 10.1371/journal.pcbi.1012870 (PMC11957563; doi:10.1371/journal.pcbi.1012870)

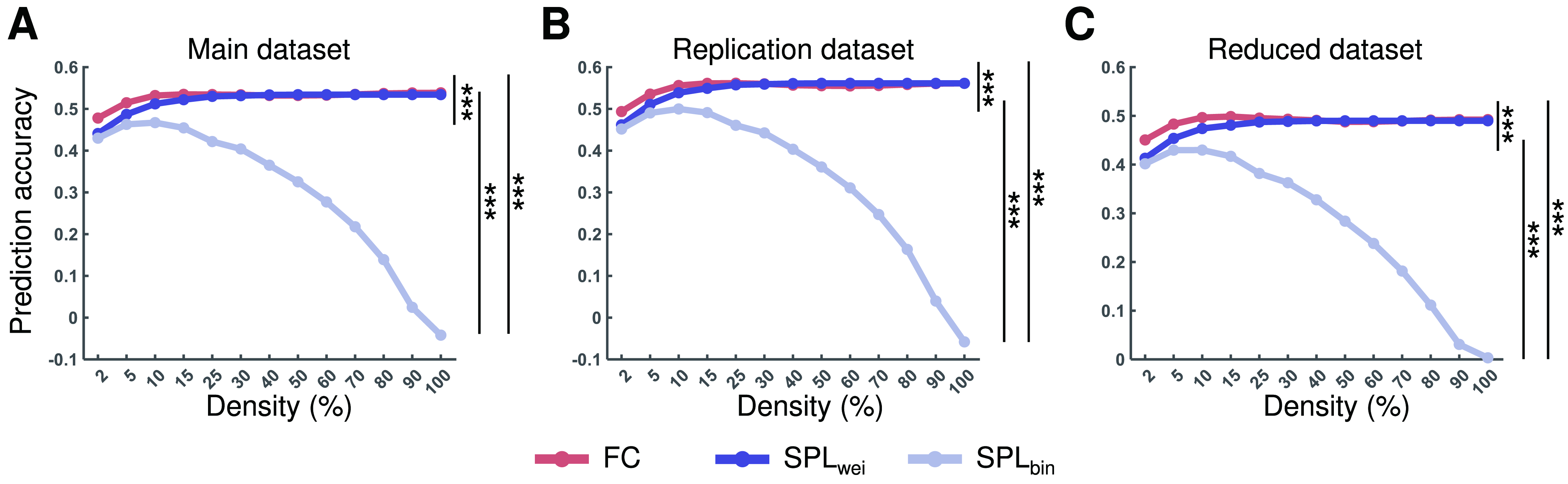

Supplement: S1 Fig — Statistical comparisons were performed for the main (A), replication (B), and reduced (C) datasets. Statistical significance was identified based on the area under the curve (AUC) across all density thresholds. FC, functional connectivity; SPLwei, shortest path length based on weighted network; SPLbin, shortest path length based on binary network. ***p < 0.001 (p < 0.05, Bonferroni corrected). (TIF) [file pcbi.1012870.s001.tif]

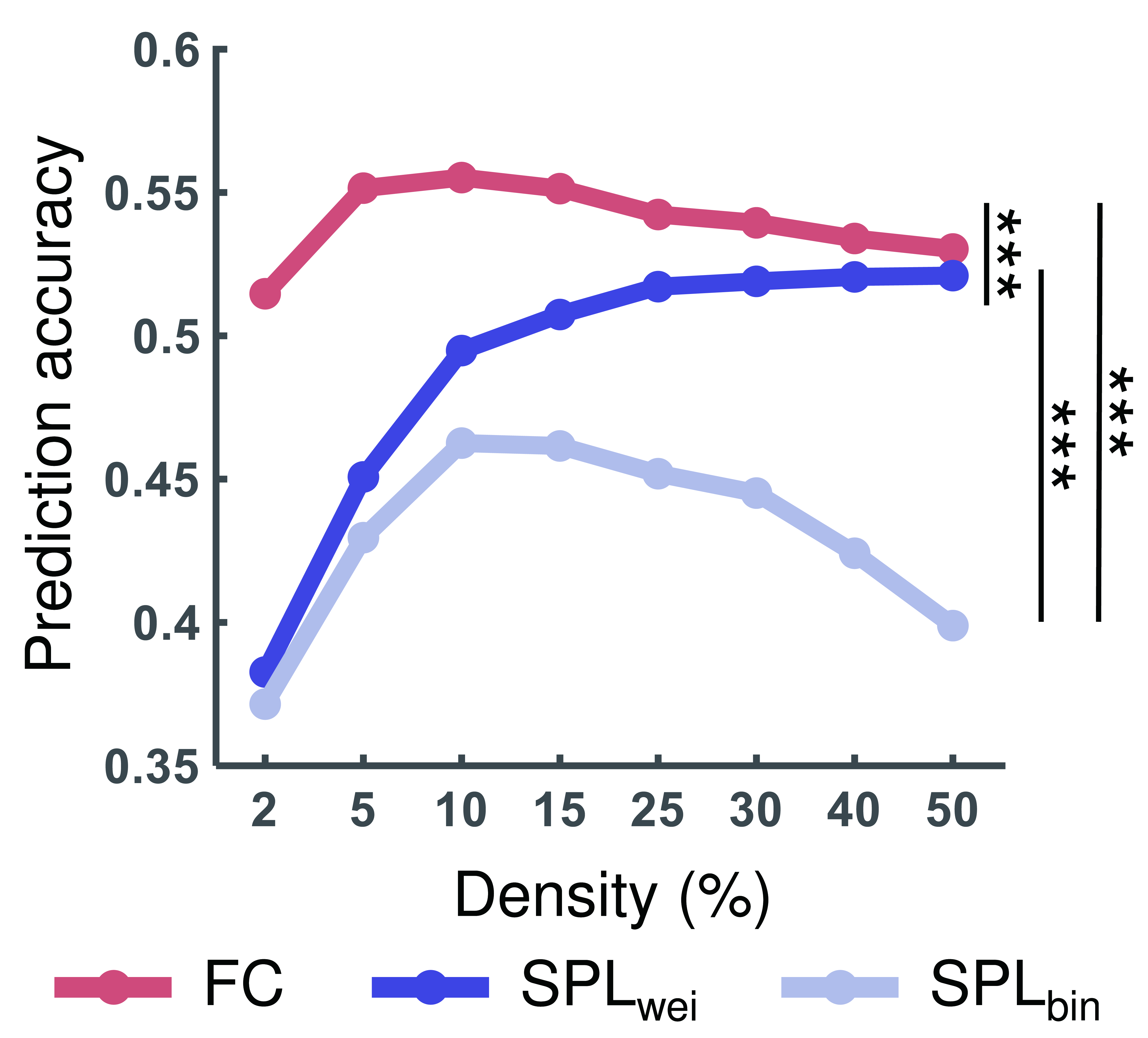

Supplement: S2 Fig — Statistical significance was identified based on the area under curve (AUC) across all sparsity thresholds. FC, functional connectivity; SPLwei, shortest path length based on weighted network; SPLbin, shortest path length based on binary network; and ***p < 0.001 (p < 0.05, Bonferroni corrected). (TIF) [file pcbi.1012870.s002.tif]

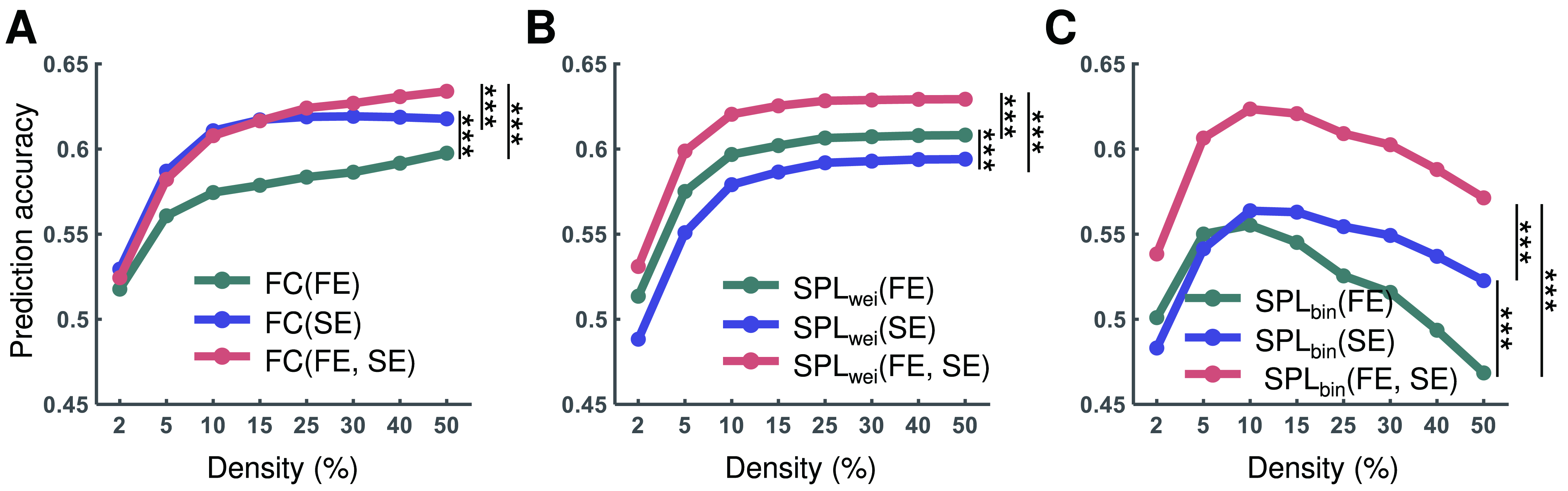

Supplement: S5 Fig — Accuracy of the activity flow prediction based on FC (A), SPLwei (B), and SPLbin (C) with different embeddings. Statistical significance was identified based on the area under curve (AUC) across all sparsity thresholds. FC, functional connectivity; FE, functional embedding; SE, spatial embedding; SPLwei, shortest path length based on weighted network; SPLbin, shortest path length based on binary network; ns, nonsignificant. ***p < 0.001 (p < 0.05, Bonferroni corrected). (TIF) [file pcbi.1012870.s005.tif]

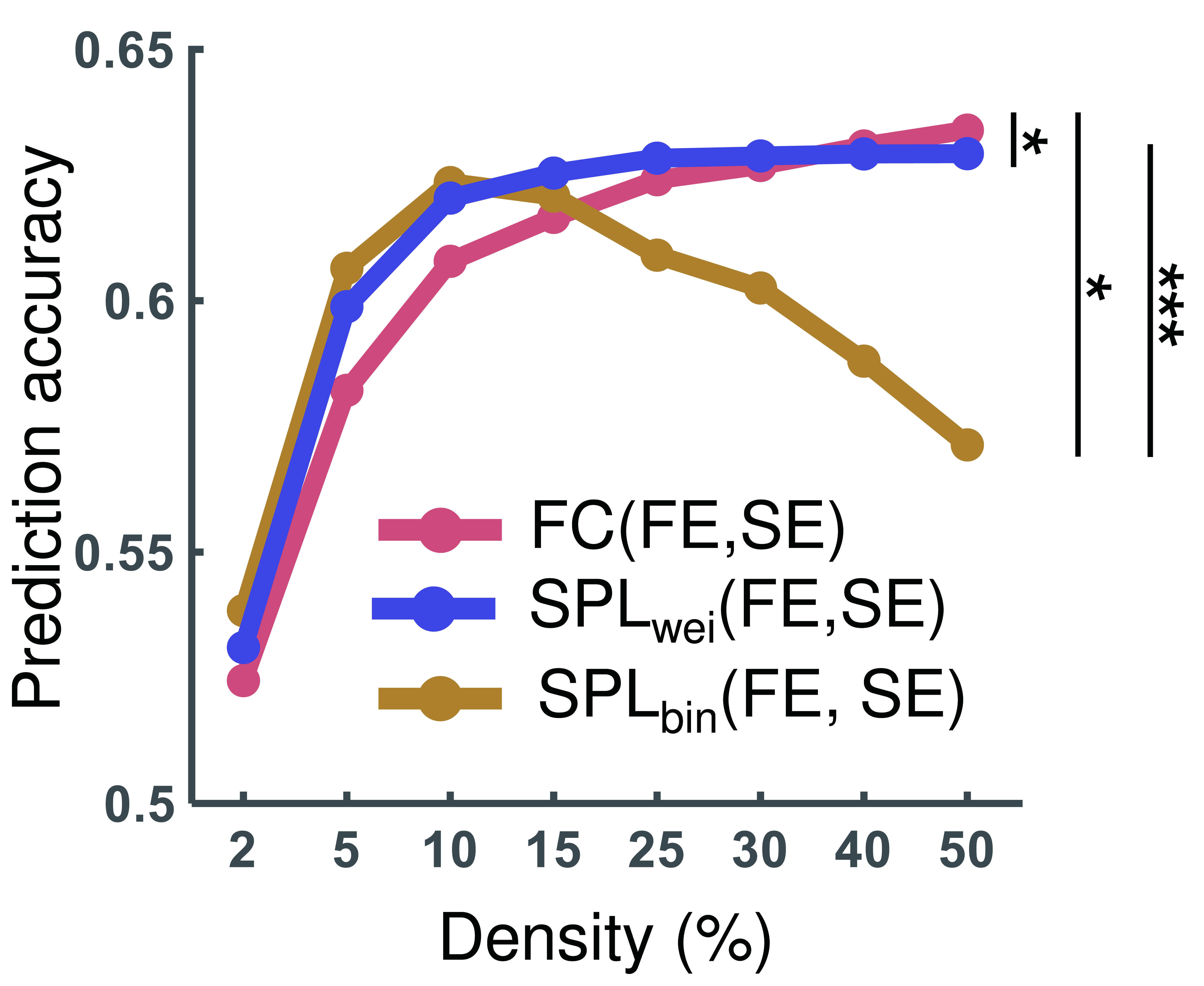

Supplement: S6 Fig — Statistical significance was identified based on the area under curve (AUC) across all sparsity thresholds. FC, functional connectivity; FE, functional embedding; SE, spatial embedding; SPLwei, shortest path length based on weighted network; SPLbin, shortest path length based on binary network; ns, nonsignificant. *p < 0.05, ***p < 0.001 (p < 0.05, Bonferroni corrected). (TIF) [file pcbi.1012870.s006.tif]

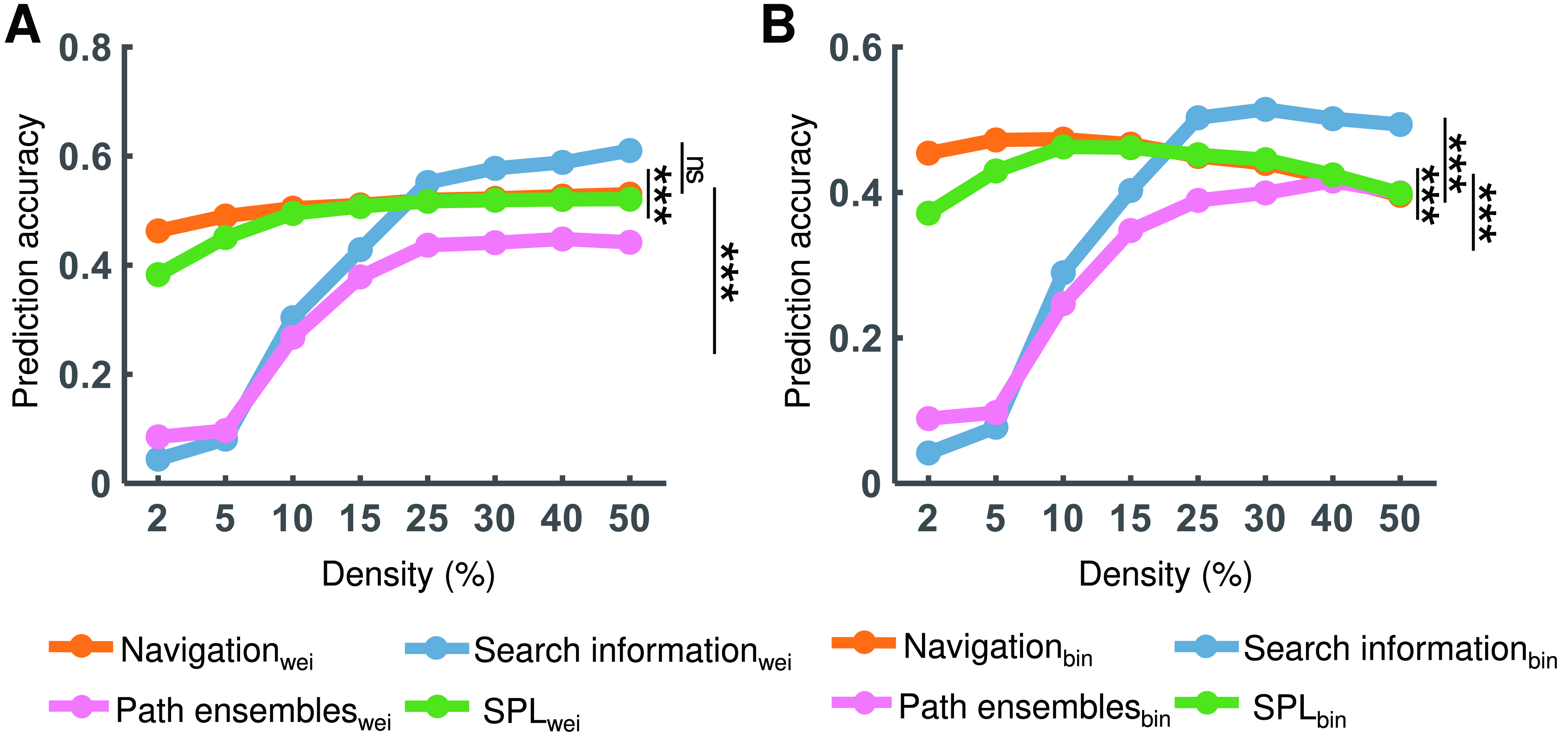

Supplement: S8 Fig — Statistical significance was identified based on the area under curve (AUC) across all sparsity thresholds. Xwei, routing metric X calculated based on the weighted network; Xbin, routing metric calculated based on the binary network. ***p < 0.001 (p < 0.05, Bonferroni corrected). (TIF) [file pcbi.1012870.s008.tif]

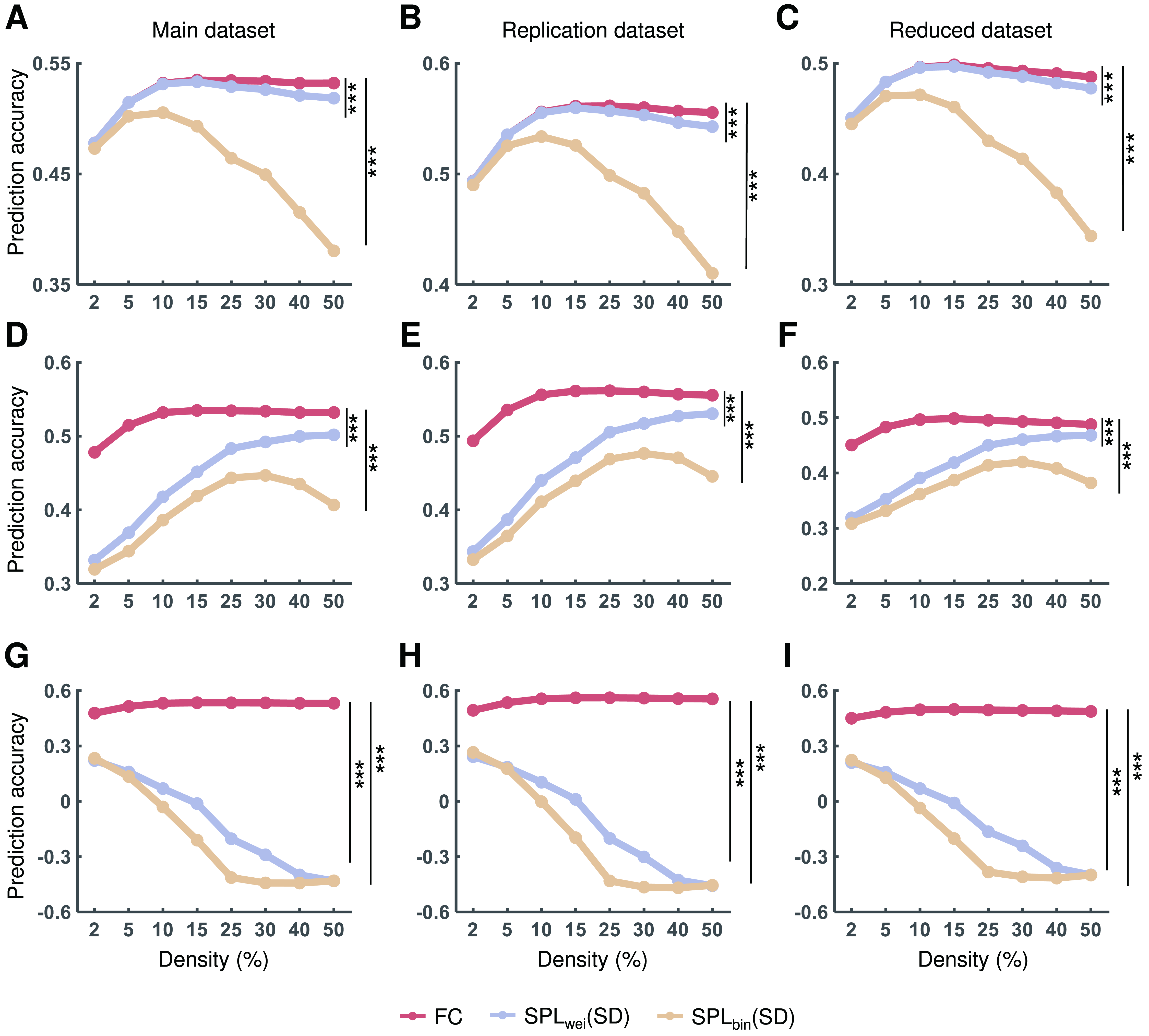

Supplement: S9 Fig — To keep the same density with the original FC network, SPL networks were sparsified according to path weights directly (A-C), with direct paths and indirect paths preserved proportionally (D-F), and with only indirect paths preserved (G-I). Statistical significance was identified based on the area under the curve (AUC) across all density thresholds. FC, functional connectivity; SPLwei(SD), shortest path length based on weighted network and having the same density with the original FC network; SPLbin(SD), shortest path length based on binary network and having the same density with the original FC network. ***p < 0.001 (p < 0.05, Bonferroni corrected). (TIF) [file pcbi.1012870.s009.tif]
