# Supplementary material for: Exploring the transmission of cognitive task information through optimal brain pathways
Source: PLoS Comput Biol. 2025 Mar 7;21(3):e1012870. doi: 10.1371/journal.pcbi.1012870 (PMC11957563; doi:10.1371/journal.pcbi.1012870)
Supplement: Attachment — Submitted filename: Response to Reviewers.docx [file pcbi.1012870.s011.docx]

**Reviewer #1**

**Summary and merits:**

**The authors provide a comprehensive overview of the prediction accuracy of different network definitions on activity flow mapping. The main results appear to demonstrate that direct path (defined as Pearson’s FC) is more accurate than other, indirect methods such as shortest path length. They test their results over a range of sparsity values for the original FC matrix and further demonstrate that the incorporation of spatial distance and functional asymmetry improve prediction accuracy. The manuscript is well written and the results are presented clearly. I do have a few comments, below.**

**Response:** We thank the reviewer for the detailed and positive evaluation for our manuscript and constructive comments, to which we respond below.

**Comments:**

**1. The reported methods on the activity flow paradigm are poorly reported. From the manuscript, it is not clear what Ai represents (e.g. beta-weights?) and there is no mention of** **the leave-one-region-out simulations that I assume must take place for the comparison between prediction and actual activity to be meaningful.**

**Response:** Thanks for your valuable comment. *A_i_* represents actual activation (i.e., beta-weights or difference in beta-weights between two conditions) for region *i* in a given task, which is estimated by general linear model. Yes, activity flow model is implemented through leave-one-region-out simulations. Then, the similarity (*r*) is computed by Pearson’s correlation between prediction and actual activity. We have clarified this in the Methods section.

**2. My major criticism of the framework presented deals with the sparsity of the compared networks. The authors define sparsity for their direct-path network (FC). From this sparse FC network, they then compute their other (in-direct) networks (e.g. SPLwei/SPLbin). Because the in-direct networks consider in-direct connections, the sparsity of the resulting SPL networks decreases (see Figure 2). It seems to me that across all sparsity levels tested, the SPLwei networks will be nearly fully connected in each case, and as** **the sparsity of the original FC network is increased, they will be generated from networks with higher signal-to-noise ratio. Thus it makes sense that** **the prediction accuracy of the SPLwei networks will continue to increase, even while the FC begins to plateau, as the SPL edges are less polluted by erroneous connections (see Figure 3). In fact, it appears possible that if the authors extended their analysis beyond sparsity levels of 0.25, SPLwei may overtake FC in prediction accuracy. I believe that the manuscript would benefit from (a) extension of sparsity beyond 0.25 to see if this is the case, (b) discussion of the above limitations, and (c) an analysis that actually keeps the sparsity of the resulting networks consistent by applying the same sparsity thresholds to the indirect networks.**

**Response:** Thanks for your insightful suggestions. For graph theory analysis, previous studies often treated the human brain as a sparse network. Since there is currently no definitive criterion for selecting a single threshold, a range of density thresholds (e.g., 2%-25%) is commonly used (Achard & Bullmore, 2007; Bassett et al., 2008). The SPL networks are denser than the original FC network, which is attributed to the indirect paths considered. The reviewer speculates that SPL_wei_ may overtake FC in prediction accuracy if extending the analysis beyond density levels of 25%. The reason is that the SPL edges are less polluted by erroneous connections, when the density of the original FC network is increased.

Following the reviewer’s suggestion, we have extended network density to 100% to examine this potential effect. However, to keep consistency with traditional brain network analysis, our main results were updated with an extension of the network density to 50%. We found that the prediction accuracy of both the FC and SPL_wei_ networks began to plateau when network density beyond 25% (revised Fig. 3 and Fig. S1). This result indicates that SPL_wei_ does not overtake FC in prediction accuracy when extending the density threshold beyond 25%. Instead, this result suggests that indirect paths may play adverse effects on prediction accuracy, when the original FC network is sparse. As network density increases, many indirect paths may be replaced by direct paths, making the prediction accuracy comparable. We have updated the related analyses and results with an extension of network density and also added the above discussion in the revised manuscript.

In addition, we have conducted an analysis that keeps the density of the resulting networks consistent by applying the same density thresholds to the SPL networks. Here, we considered three situations as follows: (1) a sparsification of SPL networks according to path weights directly. We found that the prediction accuracy with the shortest path was also lower than that with the direct path, and this difference mainly occurred in the case of high network density (revised Fig. S9). It is possible that the removed SPL edges are mainly indirect paths for low network density but direct paths for high network density, because many indirect paths may be replaced by direct paths as the network density increases. (2) a sparsification of SPL networks with direct and indirect paths preserved proportionally. The prediction accuracy with the SPL networks was dramatically lower than that with the FC network across all density thresholds (revised Fig. S9). (3) a sparsification of SPL networks with only indirect paths preserved. We found that the prediction accuracy with the SPL networks was dramatically lower than that with the FC network across all density thresholds (revised Fig. S9). These findings suggest that the overall performance of activity flow prediction is determined by routing protocols rather than network density. We have added the above analyses, results, and discussion in the revised manuscript.

**Revised Fig. S1** Accuracy of the activity flow prediction based on the direct (FC) and shortest (SPL_wei_ and SPL_bin_) paths with an extension of the network density to 100%. Statistical comparisons were performed for the main (a), replication (b), and reduced (c) datasets. Statistical significance was identified based on the area under the curve (AUC) across all density thresholds. FC, functional connectivity; SPL_wei_, shortest path length based on weighted network; SPL_bin_, shortest path length based on binary network. ****p* < 0.001 (*p* < 0.05, Bonferroni corrected).

**Revised** **Fig. 3** Accuracy of the activity flow prediction based on the direct (FC) and shortest (SPL_wei_ and SPL_bin_) paths. Statistical comparisons were performed for the main (a), replication (b), and reduced (c) datasets. Statistical significance was identified based on the area under the curve (AUC) across all density thresholds. FC, functional connectivity; SPL_wei_, shortest path length based on weighted network; SPL_bin_, shortest path length based on binary network. ****p* < 0.001 (*p* < 0.05, Bonferroni corrected).

**Revised Fig. S9** Effects of network density on activity flow prediction for different routing protocols. To keep the same density with the original FC network, SPL networks were sparsified according to path weights directly (a-c), with direct paths and indirect paths preserved proportionally (d-f), and with only indirect paths preserved (g-i). Statistical significance was identified based on the area under the curve (AUC) across all density thresholds. FC, functional connectivity; SPL_wei_(SD), shortest path length based on weighted network and having the same density with the original FC network; SPL_bin_(SD), shortest path length based on binary network and having the same density with the original FC network. ****p* < 0.001 (*p* < 0.05, Bonferroni corrected).

**Reviewer #2:**

**Summary:**

**In “Exploring the transmission of cognitive task information through optimal brain pathways”, Wang et al. build on the activity flow framework to investigate the graph routing protocols underlying the transmission of cognitive information in the human brain. The study is highly original and builds bridges between typically separate areas of neuroscience, such as graph theory and cognitive task brain activations. The general approach is highly promising, yet there are some flaws with the conceptualization of the activity flow modeling procedures. See below for details, but to summarize, the main issue is that routing protocols are allowed to modify connectivity estimates, violating basic physical principles for how activity flows propagate in actual brains. For example, shortest paths are calculated based on standard walks over FC graphs (much as flows actually occur in the brain), but then the estimated shortest path lengths are used to create new connections weighted by those paths. It is unclear whether the authors think these new connections actually exist, or whether they are representing something more abstract than actual new connections. In any case, the results are difficult to comprehend from a simple activity propagation perspective, due to graph measures on a given graph actually modifying that graph for subsequent activity propagation calculations. Instead, I suggest the authors consider keeping the graph stable and simulate flows according to the given communication protocol (e.g., shortest path) being tested.**

**Response:** We thank the reviewer for the detailed and positive evaluation for our manuscript and constructive comments, to which we respond below.

**Major concerns:**

**1. It is unclear what “sparsity” means in the plots. Are there fewer connections with more “sparsity” in the plots, or fewer? Often density is used (rather than sparsity) for thresholding, so I want to make sure sparsity was really meant (rather than density). I assume “density” is meant whenever the term “sparsity” was used. For instance, 0.15 is used as an example sparsity in the text, which would correspond (if 0.15 is to be converted to a percentage) to a density of 85% (1-0.15=0.85*100). That is quite dense. Also, it seems that** **the density (or sparsity) values should be reported in terms of percentages (rather than decimal point values).**

**Response:** Thanks for pointing out this issue and also for your kind suggestion. In our original manuscript, sparsity (e.g., 0.02) was actually meant to density (e.g., 2%). To avoid this confusion, we have used density rather than sparsity for network thresholding in the revised manuscript. In addition, the density values are reported in terms of percentages rather than decimal point values.

**2．The biggest issue I see with this study is that the main analysis (Figure 1) assumes a difference** **between a “direct” flow route and a “shortest path” flow route, while those are often the same thing. The authors appear to** **force a difference between “direct” and “shortest path”, but this just makes their “shortest path” no longer the true shortest path. Ultimately, this sets up a straw man argument, wherein the “shortest path” communication protocol is destined to fail. Why exclude the true shortest path (the direct path) when considering the shortest path?**

**Response:** We apologize for making this confusion. For Figure 1, we just want to show an example for a “direct” flow route and a “shortest path” flow route. Yes, those routes are often the same thing. In our main analyses, we do not exclude the true shortest path (the direct path) when considering the shortest path. Actually, the “shortest path” communication protocol may include both the direct path and multi-step shortest path. To avoid this confusion, we have modified Figure 1 for a better illustration of the difference between a “direct” flow route and a “shortest path” flow route. Moreover, we have emphasized that we do not exclude the true shortest path (the direct path) when considering the shortest path in the Methods section.

**Revised Fig. 1** Schematic diagram of two types of typical neural communication routes (i.e., single-step and multistep). (a) Illustration of the direct path (single-step) from the source node *s1* to the target node *t* (red dash line) and the shortest path (multistep) from nodes *s2* to *t* (green dash line). The brain render was drawn by hand using Microsoft Visio. (b) Illustration of activity flow modeling with different routing strategies. *Route_st_* indicates direct functional connectivity, shortest path, or other network communication strategies (i.e., navigation, path ensembles, and search information) between nodes *s* and *t*.

**3. However, careful consideration of Figure 2b (and the Methods) suggests the “shortest path” may be something more complex, with the direct paths and multi-step paths all contributing simultaneously. This is confusing, as Figure 1 illustrated a more circumscribed definition of “direct” and “shortest path”. Instead of having all regions’ activity making a weighted contribution to the target,** **it seems that activity flows should be simulated as actual flow processes, rather than simply modifying the FC/weight values. While clever, only modifying the FC values (rather than simulating alternate flow routes) confuses direct and alternate-route processes. Perhaps most problematically, all sources are included (using a leave-one-out approach), making it such that the direct path should always be the best routing protocol. This is because only direct paths actually (in reality, outside the models) have access to the target, with all other signals needing to pass through the direct sources. What is needed is likely a different activity flow routing setup, such as is used in several recent studies (see Ito T, Yang GR, Laurent P, Schultz DH, Cole MW (2022)** **Constructing neural network models from brain data reveals representational transformations linked to adaptive behavior. Nat Commun 13:673. And Cocuzza CV, Sanchez-Romero R, Ito T, Mill RD, Keane BP, Cole MW (2024)** **Distributed network flows generate localized category selectivity in human visual cortex Kay K, ed. PLoS Comput Biol 20:e1012507.). These studies start with a subset of activations, then** **simulate activity flows over multiple steps to test the efficacy of multi-step activity flow processes. In contrast, the current study’s approach simulates flow processes as if they were all direct, simply adding a connection from a source to a target with a weight reflecting the length of a shortest path from that source to that target. This violates the organization of the estimated connectivity graphs, which requires that indirect signals must pass through direct sources on the way to a given target. That said, there may be some clever way to think about the problem that does not require simulations that are physically realistic (in the sense of only direct sources directly impacting targets), but if so this should be made much clearer in the manuscript.**

**Response:** Thank you for the valuable suggestion. Yes, the “shortest path” may include the direct paths and multi-step paths all contributing simultaneously. We have modified Figure 1 following the comment above. In our original analyses, we simply added a connection from a source to a target with a weight reflecting the length of a shortest path from that source to that target. As the reviewer mentioned, it seems that activity flows should be simulated as actual flow processes. Inspired by recent studies (Cocuzza et al., 2024; Ito et al., 2022), we have simulated activity flows over multiple steps to test the efficacy of multi-step activity flow processes. Specifically, we performed stepwise calculation for the transformation of cognitive task activation from source to target according to the shortest path. As a consequence, activity flow prediction based on the stepwise shortest path simulation was formulated as follows:

$$E_{t}=\sum_{s\neq t\in V} A_{s}\prod_{u,v\in\Omega_{s\longrightarrow t}} \frac{1}{T_{uv}}$$

Where $\Omega_{s\longrightarrow t}=\{s, i, j, \ldots,k, t\}$ denotes the corresponding nodes sequence of the shortest path from source node *s* to target node *t*. $E_{t}$ is the predicted activation for the node *t*. $A_{s}$ denotes actual activation of region *i* for a given task. $T_{uv}=\{T_{si},T_{ij},\ldots,T_{kt}\}$ is the topological distance along the shortest path nodes sequence from *s* to *t*.

We found that the prediction accuracy with stepwise activity flow processes along the shortest path was significantly lower than that with the shortest path length directly (revised Fig. 4). This result suggests that the shortest path length routing is superior to the stepwise shortest path protocol in cognitive information transmission.

In analogy with artificial neural networks, recent studies have modeled activity flows over multiple steps (Cocuzza et al., 2024; Ito et al., 2022). However, these models mainly focus on specific nervous systems (e.g., visual processing system), which simulate decentralized information processing. In contrast, the shortest path routing requires the knowledge of global network topology. While progressive information propagation seems biologically plausible, it may be not the case for the shortest path routing at the level of whole brain. On the other hand, many graph measures based solely on the shortest path length can well characterize brain hubs that play a critical role in information communication underlying diverse cognitive functions (van den Heuvel and Sporns, 2013). Therefore, it may be appropriate to use the shortest path length as a weight for routing cognitive information transmission. We have added the above analysis, result, and discussion in the revised manuscript.

**Revised Fig. 4** Accuracy of the activity flow prediction based on the shortest (SPL_wei_) and stepwise shortest (SP_wei_) paths. Statistical comparisons were performed for the main (a), replication (b), and reduced (c) datasets. Statistical significance was identified based on the area under the curve (AUC) across all density thresholds. SPL_wei_, shortest path length based on weighted network; stepwise SP_wei_, shortest path identified by weighted network and simulating stepwise activity flow processes. ****p* < 0.001 (*p* < 0.05, Bonferroni corrected).

**4. The results in Figure 3 suggest that the results are highly dependent on network density thresholds. In this case at a 0.24 density the direct and shortest path results converged. In general, many results are dependent on network density, raising concerns about the robustness of the results.**

**Response:** Thanks for raising this concern. The aim of this study is to bridge the gap between graph-theory-based brain network analysis and cognitive task activation. For graph theory analysis, previous studies often treated the human brain as a sparse network. Since there is currently no definitive criterion for selecting a single threshold, a range of density thresholds (e.g., 2%-50%) is commonly used (Achard & Bullmore, 2007; Bassett et al., 2008). In general, graph measures and differences between groups (e.g., patients versus controls) may vary with network density (Achard & Bullmore, 2007; He et al., 2008). Consistently, we found that the direct and shortest path results converge at the high density threshold. Theoretically, as the network density increases, many indirect paths may be replaced by direct paths, making the prediction accuracy comparable. We have clarified this in the Discussion section.

**Minor concerns:**

**1. It is stated that “the shortest path routing is unrealistic for decentralized nervous systems because it requires individual elements to have knowledge of the global network topology”. However, Misic et al. (2015) (Mišić B, Betzel RF, Nematzadeh A, Goñi J, Griffa A, Hagmann P, Flammini A, Ahn Y-Y, Sporns O. 2015. “****Cooperative and Competitive Spreading Dynamics on the Human Connectome”. Neuron. 86:1518–1529.) showed that shortest paths are special even** **in a decentralized system because signals arrive first via shortest paths even with simple diffusion processes. Thus, shortest paths are not properly characterized here.**

**Response:** Thanks for your valuable comment. This statement was originally drawn from the emerging evidence that challenges the shortest path assumption. For instance, several network communication models (e.g., search information, path ensembles, and navigation) that do not require centralized knowledge have been proposed, which usually enable better inference of structure-function correspondences. As the reviewer mentioned, Misic et al. have shown that shortest paths are special even in a decentralized system. Therefore, we have revised the statement in the Introduction section as follows:

“*……Computational modeling work has also shown that the shortest path structure of the human connectome accelerates the spread of information cascades [41]. However, the shortest path routing has been controversial for the theoretical models of brain function because it requires a biologically implausible assumption that neural elements have knowledge of global network topology [42, 43]. Accordingly, several network communication models that do not require centralized knowledge, such as diffusion models [44-47], path ensembles [48], and navigation [49], have been proposed to characterize neural signaling. Although these network communication strategies usually enable better inference of structure-function correspondences, little is known about whether they are superior to the shortest path routing for diverse cognitive information transmission.*”

**2. All negative connections were set to 0 because of “the lack of explicit meaning”. It would be important to make it clear what is meant here, since negative connections do indeed have a meaning in theory (inhibitory connectivity).**

**Response:** Thanks for raising this concern. Negative connections may have a meaning in theory (inhibitory connectivity). However, it remains to be demonstrated whether the negative connections estimated by fMRI represent inhibitory connectivity. Therefore, previous brain network studies usually set negative connections to 0. Following previous studies (Rubinov & Sporns, 2010; Zhu et al., 2023), we also set all negative connections to 0. We have clarified this in the Methods section.

**3.** **Use of Pearson correlation is a problem, as a “direct” route has a high probability of not existing. See** **Reid AT, Headley DB, Mill RD, Sanchez-Romero R, Uddin LQ, Marinazzo D, Lurie DJ, Valdés-Sosa PA, Hanson SJ, Biswal BB, Calhoun V, Poldrack RA, Cole MW. 2019. “****Advancing functional connectivity research from association to causation”. Nat Neurosci. PMID: 31611705. It would be important to acknowledge limitations of using Pearson correlation as a functional connectivity measure.**

**Response:** Thanks for your kind suggestion. While Pearson correlation is a commonly used method for measuring FC, it may produce spurious connections and cannot characterize causal interactions (Reid et al., 2019), which could affect the inference of activity flows. We have acknowledged this limitation in the revised manuscript as follows.

“*Second, while Pearson correlation is a commonly used method for measuring FC, it may produce spurious connections and cannot characterize causal interactions [80], which could affect the inference of activity flows. A recent study used the Bayesian network-based Peter-Clark algorithm to infer directed and causal activity flow routes [20]; alternative directed FC approaches can also be applied, such as dynamic causal models [81, 82], Granger causality [83], and transfer entropy [84]. However, to date, there are no methods that can make perfect causal inferences using noninvasive neuroimaging techniques, and the causal connectivity inferred by different methods can be inconsistent [85-87].*”

“*Finally, several FC methods have been proposed to improve activity flow prediction [15, 20]. However, in this study, we used the standard Pearson’s correlation approach, which requires few assumptions and is most frequently employed for the construction of functional brain networks. Thus, it enables better linking of the activity flow mapping framework and graph theoretical framework.*”

**4. It would be important to see the accuracies of the activity flow predictions in the text, so the reader can better interpret the results. It would also be helpful to see the accuracies in terms of r-values, rather than AUC, since r-values are easier to understand.**

**Response:** Thanks for your kind suggestion. Yes, it is helpful to see the accuracies in terms of *r* values. However, the accuracies of the activity flow predictions were calculated over a wide range of network density. It seems not suitable for reporting all *r* values in the text (actually all *r* values are shown in Figures). Based on strategies commonly used in brain network studies, we performed statistical analysis for the AUC, which provides a summarized measure independent of a single threshold selection (He et al., 2008; Lei et al., 2015). Following the reviewer’s suggestion, we have added the range of *r* values over all density thresholds for main results in the text. An example is shown below.

“*For main dataset, we found that the mean prediction accuracies ranged from r = 0.48 to r = 0.53 for the direct path,* *from r = 0.44 to r = 0.53 for the SPL_wei_, and from r = 0.33 to r = 0.47 for the SPL_bin_ over all density thresholds. For replication dataset, we found that the mean prediction accuracies ranged from r = 0.49 to r = 0.56 for the direct path, from r = 0.46 to r = 0.56 for the SPL_wei_, and from r = 0.36 to r = 0.50 for the SPL_bin_ over all density thresholds. For reduced dataset, we found that the mean prediction accuracies ranged from r =0.45 to r = 0.50 for the direct path, from r = 0.41 to r = 0.49 for the SPL_wei_, and from r = 0.28 to r = 0.43 for the SPL_bin_ over all density thresholds.*”
